# A Locked Nucleic Acid Probe Based on Selective Salt-Induced Effect Detects Single Nucleotide Polymorphisms

**DOI:** 10.1155/2015/391070

**Published:** 2015-08-11

**Authors:** Jing Zhang, Huizhe Wu, Qiuchen Chen, Pengfei Zhao, Haishan Zhao, Weifan Yao, Minjie Wei

**Affiliations:** Department of Pharmacology, School of Pharmacy, China Medical University, Liaoning Key Laboratory of Molecular Targeted Anti-Tumor Drug Development and Evaluation, No. 77 Puhe Road, Shenbei District, Shenyang 110013, China

## Abstract

Detection of single based genetic mutation by using oligonucleotide probes is one of the common methods of detecting single nucleotide polymorphisms at known loci. In this paper, we demonstrated a hybridization system which included a buffer solution that produced selective salt-induced effect and a locked nucleic acid modified 12 nt oligonucleotide probe. The hybridization system is suitable for hybridization under room temperature. By using magnetic nanoparticles as carriers for PCR products, the SNPs (*MDR1* C3435T/A) from 45 volunteers were analyzed, and the results were consistent with the results from pyrophosphoric acid sequencing. The method presented in this paper differs from the traditional method of using molecular beacons to detect SNPs in that it is suitable for research institutions lacking real-time quantitative PCR detecting systems, to detect PCR products at room temperature.

## 1. Introduction

Single nucleotide polymorphisms (SNPs) are a DNA sequence polymorphism induced by single nucleotide mutation within the genome. It is one of the most common types of human inheritable mutation [1]. Some of the SNPs within the DNA not only interfere with the occurrence and development of multiple types of disease, but also can be used as potential diagnostic or prognostic molecular markers; therefore, they play a more and more important role in the early diagnosis or prognostic surveillance for personalized and targeted therapies in patients [2, 3]. One of the most common methods is to use oligonucleotide probes to detect SNPs at known loci. Molecular beacons (MBs) are a type of oligonucleotide probe with stem-loop structure, which has demonstrated high specificity in SNPs detection [4, 5]. Due to the high complexity involved in the design of MBs, which usually requires the aid of a real-time quantitative PCR detecting system, the cost of testing is very high. A new testing method with highly specific, easy-to-design probes, without requiring an equipment for temperature control, is highly advantageous.

There were reports of ΔTm values reaching 26°C, for the double chain formed from the hybridization between LNA modified 8 nt probes with complementary templates and SM templates [6]. LNA is an oligonucleotide analog in which the 2′-oxygen and the 4′-carbon of ribose moiety are joined via O-methylene (oxy-LNA) bridge, S-methylene (thio-LNA) bridge, or amino-methylene (amino-LNA) bridge. This double-loop structure of the ribose severely limits its ability to twist horizontally [7]. The LNA-DNA double helix possesses markedly higher thermostability than that of the DNA-DNA double helix, and at the same time, LNA is less likely to approach mismatched nucleotide during hybridization with wrong-pairing DNA; therefore, LNA demonstrates strong ability to recognize wrong base pairings [8]. So, we propose that, within tailored buffer solutions, LNA modified short chain oligonucleotide probes can rapidly detect SNPs under room temperature without being affected by temperature fluctuation.

The* MDR1* gene is a type of genes that demonstrates multidrug resistance [9], of which the C3435T/A SNP is closely related to the occurrences of multidrug resistance in cancer cells [10], and this particular locus has been selected as the target for analysis in this study.

## 2. Materials and Methods

### 2.1. High Resolution Melting (HRM)

The length of LNA modified oligonucleotide probe was 12 nt, purchased from EXIQON (Holland). The probe C had the sequence of 5′-CTCACG^L^ ATCTCT-3′ and the probe A had the sequence of 5′-CTCACA^L^ ATCTCT-3′. The bases of upper right L labelled are LNA in the probes. The templates were purchased from Sangon bioengineering Co., Ltd. (Shanghai), with equal lengths of 107 nt. The template sequence was obtained from NCBI-SNP (http://www.ncbi.nlm.nih.gov/snp/), which was 5′-ACATTGCCTATGGAGACAACAGCCGGGTGGTGTCACAGGAAGAGAT [C/T/A]GTGAGGGCAGCAAAGGAGGCCAACATACATGCCTTCATCGAGTCACTGCCTAATGTAAGT-3′, with the SNP locus underlined. LC Green was purchased from Idaho (USA). Formamide was purchased from SIGMA. The real-time PCR with HRM (Model LightCycler 48 II) was purchased from ROCHE (Switzerland). Four types of PBS buffer with Na^+^ concentration of 1 M, 0.5 M, 0.25 M, and 0.125 M were selected, within which, the LNA probes were allowed to hybridize with the complementary template and the single-base mismatch template (MS template) under each concentration. The saturated fluorochrome LC green was added, and temperature range was set between 20 and 90°C. HRM curves were produced by real-time PCR with HRM. In the comparator arm, the LNA probe was replaced with DNA probe during hybridization with all other conditions being the same as mentioned above. When the optimal Na^+^ concentration was confirmed, different amounts of deionised formamide (10% V/V, 20% V/V, 30% V/V, and 40% V/V) were added to the hybridization process, and HRM curves were drawn.

### 2.2. Coupling of Template and Magnetic Nanoparticles

The xMag Streptavidin magnetic nanoparticles and 8-hole magnetic separator were purchased from GoldMag Nanobiotech Co., Ltd. (Xi'an); Binding buffer (TE, 1 M NaCl, and pH 7.4) was prepared; PBST (PBS with Na^+^ concentration of 0.15 M and 0.05% Tween20) was prepared. For detailed coupling procedures please refer to Zhan et al.'s work [11]. Due to the fact that the allele from gene* MDR1* C3435T/A was triallelic, 3 templates were used: CC/TT/AA, CC/TT/AA. A portion of each template was taken out to be mixed with a portion of one other template until three heterozygous templates were obtained: CT/CA/TA. These six templates represent all the subtypes of gene* MDR1* C3435T/A SNP.

### 2.3. Simulated Hybridization

The sequence and modification for Fluorescence Probe C were as follows: 5′-6FAM-CTCACG^L^ ATCTCT-3′, the sequence and modification for Fluorescence Probe T were as follows: 5′-ROX-CTCACA^L^ ATCTCT-3′, and the sequence and modification for Fluorescence Probe A were as follows: 5′-ROX-CTCACT^L^ ATCTCT-3′. Nikon florescent microscope was purchased from Japan. Fluorescence Probe C was mixed with Fluorescence Probe T with equal portions, allowed to hybrid with 6 types of templates under 20 and 30 degrees, hybridization time was 5 minutes, the purpose was to detect the allele C and T in gene* MDR1* C3435T/A. To prevent the excitation wavelength and the emission wavelength interfering with each other between different types of florescent dyes, Probe A was not modified with a third type of the fluorescence radical; instead, parallel testing was performed in a separate vessel. After hybridization was completed, PBST was used to elute any unbinding probes, magnetic separator was used to separate and discard supernatant, nanoparticles were resuspended, and 0.5 *μ*L of the mixed suspension solution was dropped onto a slide and analyzed under the florescent microscope. The 6FAM was the green florescent radical with the excitation wavelength of 494 nm, and ROX was the red florescent radical with the excitation wavelength of 584 nm. Fluorescent intensity was read, and linear range of concentration-intensity was established based on *X* (concentration) and *Y* (fluorescent intensity).

### 2.4. Testing on Real-Life Samples

The sequence and modification for the sense primer were as follows: Biotin-5′ACATTGCCTATGGAGACAACA-3′, and the sequence for the reverse primer was as follows: 5′-ACTTACATTAGGCAGTGACTCG-3′.* Pfu* DNA Polymerase expansion lab kits were purchased from Sangon Biotech (Shanghai) Co., Ltd. (Shanghai); DNA denaturing liquid was prepared; Gradient PCR machine was purchased from Takara BIO Co., Ltd. (Japan); Gene TAC 2000 Model CCD scanner was purchased from Genemic Solutions (USA). The sample type was genome DNA extracted from peripheral blood of human subjects, a total of 45 cases were supplied by the First Affiliated Hospital of the China Medical University. Firstly, PCR reactions occurred within the following PCR reaction system: 2* Pfu* DNA polymerase 25 *μ*L, genomic DNA sample 1 *μ*L, and sense primer and reverse primer with the concentration of 200 nmoL/L, 2 *μ*L each. Ultrapure water 20 *μ*L. The PCR reaction steps were as follows: denaturing: 94°C 4 minutes; annealing: 94°C 30 seconds → 55°C 30 seconds → 72°C 30 seconds, repeated 30 times; extension: 72°C 30 seconds. The PCR product had length of 107 bp, and the sequence was consistent with the template.

For the PCR products tagged with biotins, the coupling process was the same as the coupling process of the templates. Due to the high amount of samples involved, a CCD scanner with the capability of high volume testing was used.

## 3. Results and Discussion

### 3.1. Salt-Induced Effect

The phosphate backbone of the nucleotide is negatively charged, preventing any two single chain DNAs getting too close together, and the hybridization really relies on the base pairing principal, hence reducing the rate of pairing mismatch. Though Na^+^ can neutralise the negative charges of the phosphoric acid skeleton and promote the single chain DNAs to get close to each other, it can improve the rate of hybridization in turn [12]. When the Na^+^ concentration gets even higher, its ability to promote hybridization was able to overcome the repulsion effect between the phosphoric acid skeletons and it significantly improves the rate of single chain DNA hybridization; however, in the meantime, it increases the rate of wrong base pairing. When Na^+^ concentration was lower or even absent, the repulsion between phosphoric acid skeletons would be significant so that the rate of wrong base pairing would be relatively low, though the rate of hybridization between single chain DNAs would also be very slow [13]. In this study, either complementary template or single-base mismatch template with the same probe was hybridized in the same buffer, respectively. We prepared 4 types of buffer for hybridization, which had the different concentration of sodium only (0.125 M to 1.0 M). Then the HRM curves were drew by real-time PCR with HRM. Furthermore, selective salt-induced effect was observed when the concentration of sodium was 0.25 M in the buffer, which can induce hybridization reaction between two complementary single-strand DNAs, however, without the above special reaction between two single-base mismatch single-strand DNAs.

**Figure 1:**
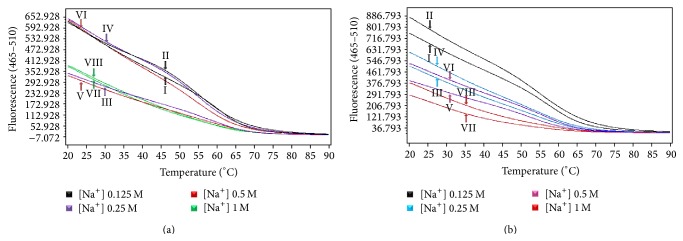
(a) The figure is showing the HRM curve for LNA probes with complementary template (complementary-curve) and the HRM curve for LNA probes with single-base mismatch template (the SM-curve). Complementary-curve (I) and SM-curve (II): in buffer with [Na^+^] 0.125 M; complementary-curve (III) and SM-curve (IV): in buffer with [Na^+^] 0.25 M; complementary-curve (V) and SM-curve (VI): in buffer with [Na^+^] 0.5 M; complementary-curve (VII) and SM-curve (VIII): in buffer with [Na^+^] 1 M. (b) The figure is showing the complementary curves and SM curves for probes without the LNA modification, and naming methods for these curves were the same as that of (a).

In Figure 1(a), we could see that, in the buffer with different Na^+^ concentrations, two types of formation of the DNA fusion curves occurred. The variations observed between the two types of fusion curves obtained depend on whether the base at the mutation positions was complementary to the probes [14]. In the buffer with suitable Na^+^ concentration, the complementary fusion curve and the SM curve from the same group demonstrated apparent “branching” effects. The complementary curves were affected by the salt-induced effect, hence showing the same formation as the fusion curves formed under high Na^+^ concentration (curves III and V). However, SM-curve was not affected by the salt-induced effect under the same Na^+^ concentration, hence showing the same formation as the fusion curves formed under low Na^+^ concentration (curves IV and VI). Therefore, we established that, when Na^+^ concentration was between 0.25 and 0.5 M, the salt-induced effect was selective and was able to improve the ability of the LNA modified probes to detect SM-templates. When we replaced LNA probes with DNA probes under the same conditions, we obtained the fusion curves shown in Figure 1(b), and the selective salt-induced effect had a significant effect on LNA modified oligonucleotide probes but did not produce evident effects on the standard oligonucleotide probes without the LNA modification. Comparing with lower concentrations of Na^+^, it was more suitable for low temperature hybridization; therefore, the reaction buffer with 0.25 M [Na^+^] was used for the subsequent study.

### 3.2. Effects of Formamide in Buffer

**Figure 2:**
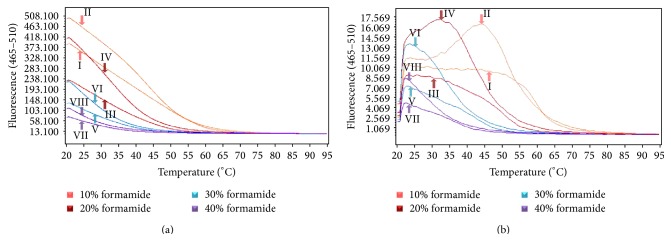
(a) The figure is showing the HRM curve for LNA probes with complementary template and SM template, when different V/V ratios of formamide were added during hybridization; complementary-curve (I) and SM-curve (II): in buffer containing 10% formamide; complementary-curve (III) and SM-curve (IV): in buffer containing 20% formamide; complementary-curve (V) and SM-curve (VI): in buffer containing 30% formamide; complementary-curve (VII) and SM-curve (VIII): in buffer containing 40% formamide. (b) The figure is showing the first-order derivative curves of (a), and naming methods for these curves were the same as that of (a).

With the addition of formamide, the Tm value of the probes was reduced, and the irregular secondary structures of the ssDNA templates were opened up, improving the chances of the probes to approach targeted sequences to complete hybridization [15]. By adding different concentration of formamide in the [Na^+^] 0.25 M buffer, we observed that the HRM curves for both the probes and templates were shifted horizontally towards lower temperature range, the amount shifted horizontally was directly proportional to the amount of formamide added, and the addition of formamide did not interfere with the Na^+^ salt-induced effect, as shown in Figure 2(a). When formamide concentration was 20%, the Tm value for the LNA-probe and SM template was 34.70°C, as shown in Figure 2(b), curve IV. It was commonly believed that when Tm value of the probes was lower than 15°C, it was more suitable for complete hybridization; therefore, 20% (v/v) formamide was selected to be used in follow-up testing.

### 3.3. Analytical Results from Fluorescent Probes

**Figure 3:**
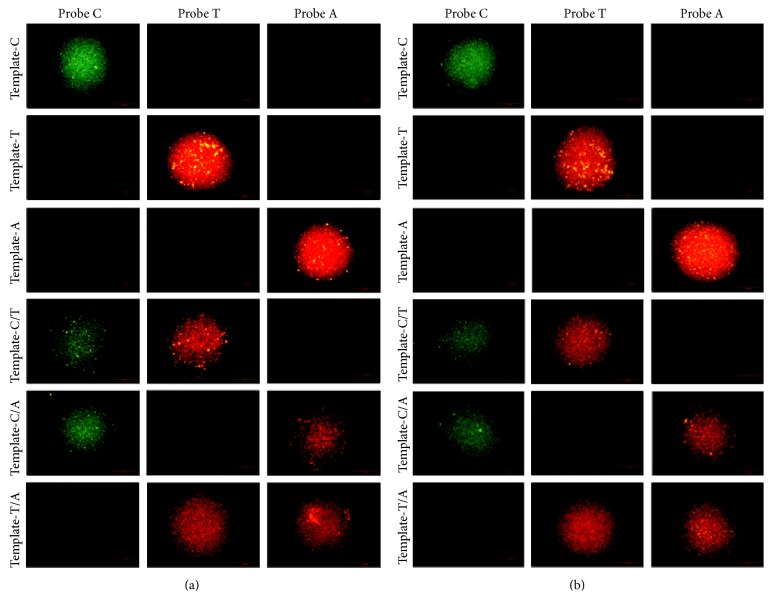
(a) Testing results for fluorescent probe under 20°C. The three pictures on each row are showing the results from the three probes to each known template. In the first row, if green fluorescent was detected, it showed that the SNP locus of* MDR1* C3435T/A gene contained allele C in the tested sample. In the middle row, if red fluorescent was detected, it showed that the SNP locus contained allele T in the tested sample; in the third row, if red fluorescent was detected, it showed that the SNP locus contained allele A in the tested sample. (b) Testing results for fluorescent probe under 30°C. The method of analysis was the same as that described in (a).

**Figure 4:**
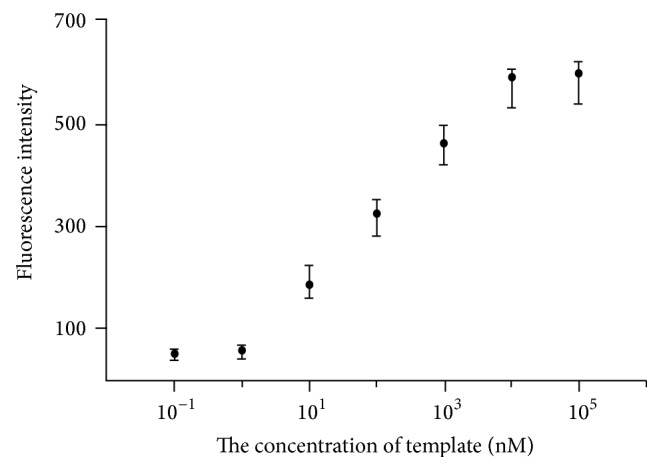
On this linear graph, coordinate axis *X* is the log of the template concentration; coordinate axis *Y* is the intensity of the green fluorescent.

Figures 3(a) and 3(b) are showing the testing results from fluorescent probes under 20°C and 30°C, respectively. It could be seen that the testing results for fluorescent probes to each types of template are identical to the known sequences. The fluorescent intensity was read, and any reading higher than background level was considered positive, linear range was 1 nM~10000 nM, and testing limit was 0.1 nmoL/L as shown in Figure 4.

**Figure 5:**
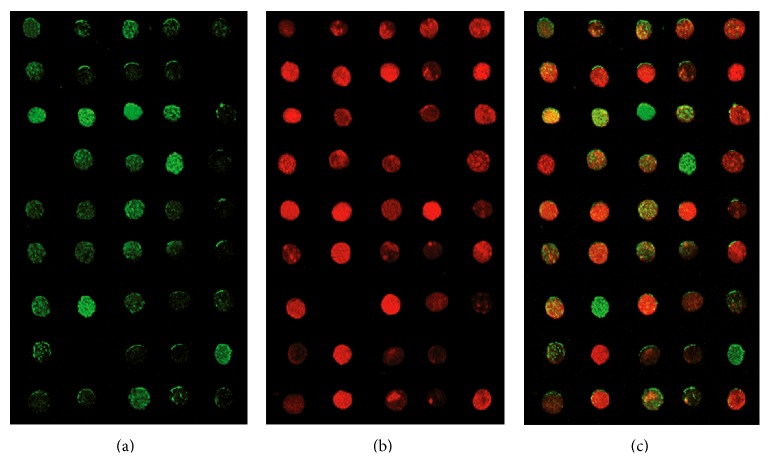
(a) The graphic results from CCD scanner under excitation wavelength of 494 nm; samples with green fluorescent carry allele C; (b) the graphic results from CCD scanner under excitation wavelength of 584 nm; samples with red fluorescent carry allele T; (c) merged graph of (a) and (b).

Lastly, we performed* MDR1* C3435T/A SNP testing on genetic samples exacted from the peripheral blood from 45 volunteers. High throughput was achieved by using fluorescent microarray scanners. From Figure 5(a), we could see that allele C was not detected in three samples, and from Figure 5(b), we could see that allele T was not detected in 4 samples, and the results were more evident in Figure 5(c) which was a merged diagram of (a) and (b). Allele A was not detected in any of the above-mentioned samples. The PCR products from all samples were sent to Shanghai Bioengineering for pyrophsphoric acid sequencing to compare with the results listed above, and conclusions were consistent. Allele A in gene* MDR1* C3435T/A in human population is rather rare [16, 17], even though this subtype was not detected in the real life samples used in this study; based on Figure 5, it could be detected by the method described in this study.

### 3.4. Advantages of Short-Chain LNA Probes

LNA has a unique double-loop structure. When hybridization occurred between LNA and wrong base pairings, its double-loop structure markedly restricted the spatial twist of the LNA ribosome; comparing with DNA probes, this noncomplementary spatial formation resulted in hybrids with relatively high thermodynamic instability; hence, LNA probe has much higher specificity against single wrong base pairings. If more modified LNA were added in the probes, the Tm value will be significantly increased [18]. The elute process can also produce great effects on the testing results. After mismatched hybridization of short chain probes, because the amount of complementary bases was relatively low, noncomplementary spatial formation produces even higher effects on the stability of hybrids, and mismatched probes could be removed with simple elution. This is one of the advantages of short chain probes; as long as specificity could be maintained, the shorter the chain, the better the probes.

## 4. Conclusion

For this hybrid system that we developed in this study for the rapid detection of SNPs under room temperature, there were three key elements involved: first, a buffer solution that was suitable for hybridization under room temperature and had the salt-induced effect that enhanced the ability of the LNA probes to detect wrong single-base pairings; second, the LNA modification of the oligonucleotide probes which significantly increased the specificity of the probes and, with the aid of the buffer, was able to detect SNPs without being affected by room temperature fluctuations; third, the relatively short oligonucleotide chain which made testing under room conditions much easier to perform, enabled the conformation of the wrongly paired LNA to produce greater effect on thermodynamic stability of the hybrids, and also utilized the characteristic of the magnetic nanoparticles to elute noncomplementary probes easily under room temperature. Single-base mismatch can be detected under room temperature of 20°C–30°C with high specificity.

## Supplementary Material

(1) “NCBI-BLAST results show that the theoretical length of PCR product is 105 bp and the sequence is the only match to target gene. Therefore, the primer design is correct, and the location of the mutations in the sequence of PCR products.”
(2) “Agarose gel electrophoresis showed that only one DNA band is located near the 100 bp DNA mark. The results indicated we have the expected DNA products by PCR.”
(3) “These 4 figures are all of 4 cases in the gene sequencing results, they represent the 3 kinds of gene mutations. Sequencing results showed no type A gene could be detected.
