# Supplementary material for: A Locked Nucleic Acid Probe Based on Selective Salt-Induced Effect Detects Single Nucleotide Polymorphisms
Source: Biomed Res Int. 2015 Aug 11;2015:391070. doi: 10.1155/2015/391070 (PMC4548076; doi:10.1155/2015/391070)
Supplement: Supplementary file 1 — (1) “NCBI-BLAST results show that the theoretical length of PCR product is 105 bp and the sequence is the only match to target gene. Therefore, the primer design is correct, and the location of the mutations in the sequence of PCR products.” (2) “Agarose gel electrophoresis showed that only one DNA band is located near the 100 bp DNA mark. The results indicated we have the expected DNA products by PCR.” (3) “These 4 figures are all of 4 cases in the gene sequencing results, they represent the 3 kinds of gene mutations. Sequencing results showed no type A gene could be detected. [file 391070.f1.pdf]

Supplementary Material

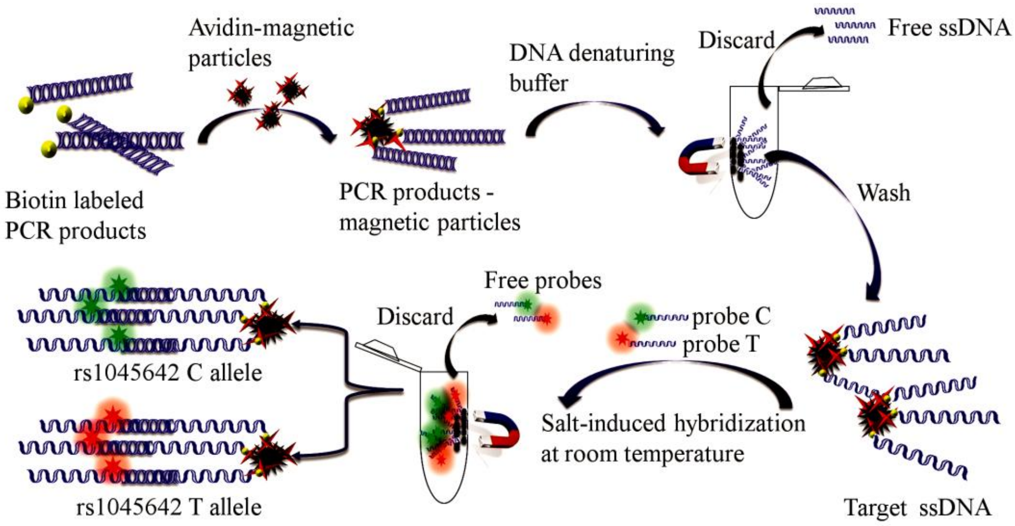

Schematic diagram . The PCR products carry biotin due to that the 5' of the forward primer was modified with biotin. PCR products were then coupled onto the surface of the nanoparticles under biotin-Avidin specificity binding. The target ssDNA were obtained by processing under the DNA denaturation solution, and under the effects of magnetic fields. Specially prepared buffer and specifically designed probes for mutational sites were added for hybridization under room temperature. If the final products were positive for green fluorescent, it showed that the rs1045642 genotype contained allele C on its locus in the tested sample; If positive for red fluorescent, it showed that the locus contained allele T.

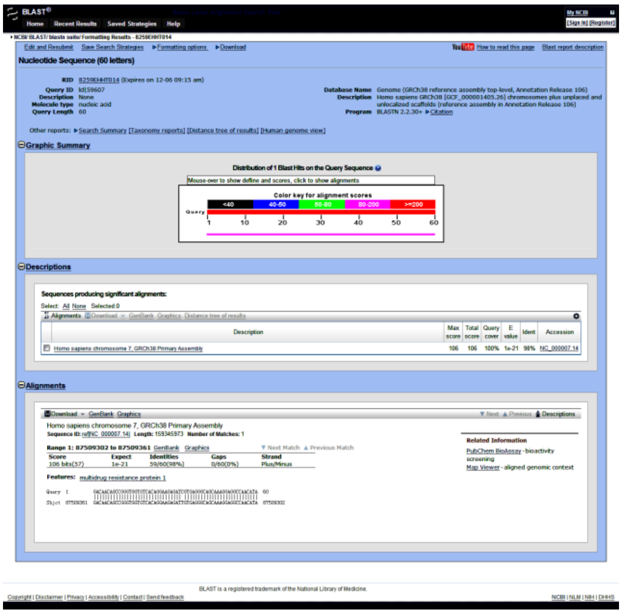

Figure 1. The sequence of PCR products were obtained from NCBI-BLAST. NCBI-BLAST results show that the theoretical length of PCR product is 105 bp and the sequence is the only match to target gene. Therefore, the primer design is correct, and the location of the mutations in the sequence of PCR products.

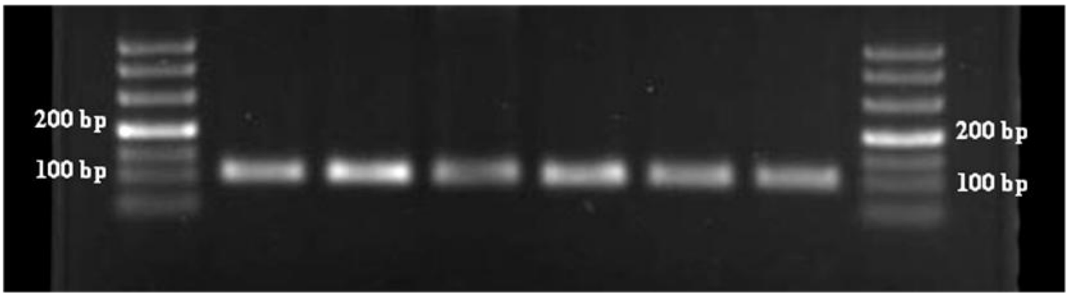

Figure 2. Electrophoregram of the PCR products.

Agarose gel electrophoresis showed that only one DNA band is located near the 100 bp DNA mark. The results indicated we have the expected DNA products by PCR.

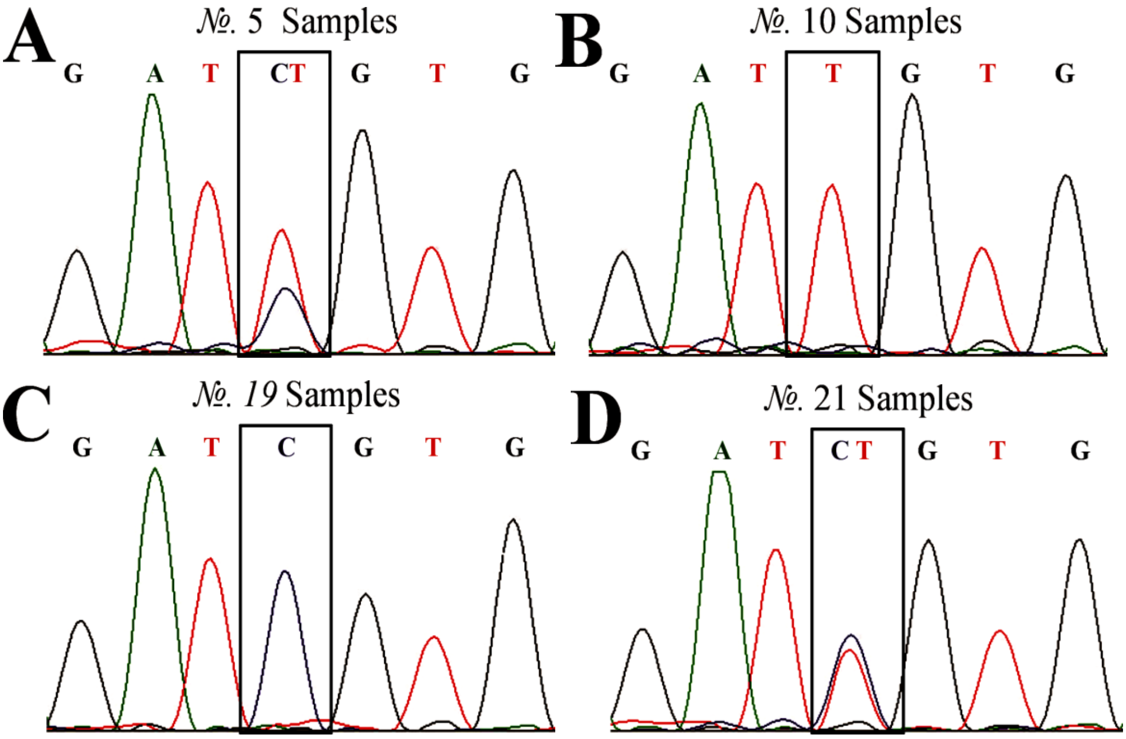

Figure 3. The partial pyrophsphoric acid sequencing results. **A**: SNP in sample 5 is C/T heterozygous; **B**: SNP in sample 10 is TT homozygous; **C**: SNP in sample 19 is CC homozygous; **D**: SNP in sample 21 is C/T heterozygous. The base within the bracket is the SNP locus of *ABCB1* C3435T/A.

These 4 figures are all of 4 cases in the gene sequencing results, they represent the 3 kinds of gene mutations. Sequencing results showed no type A gene could be detected.
